# Highly Sensitive and Transparent Strain Sensors with an Ordered Array Structure of AgNWs for Wearable Motion and Health Monitoring

**DOI:** 10.1038/s41598-019-38931-x

**Published:** 2019-02-20

**Authors:** Fanqi Yin, Huajun Lu, Hao Pan, Hongjun Ji, Shuai Pei, Hao Liu, Jiayi Huang, Jiahui Gu, Mingyu Li, Jun Wei

**Affiliations:** 1 State Key Laboratory of Advanced Welding and Joining, School of Materials Science and Engineering, Harbin Institute of Technology at Shenzhen, Shenzhen, 518055 P. R. China; 2 Center of Flexible and Printable Electronics, Harbin Institute of Technology at Shenzhen, Shenzhen, 518055 P. R. China; 3 Singapore Institute of Manufacturing Technology, 73 Nanyang Drive, 637662 Singapore, Singapore

## Abstract

Sensitivity and transparency are critical properties for flexible and wearable electronic devices, and how to engineer both these properties simultaneously is dramatically essential. Here, for the first time, we report the assembly of ordered array structures of silver nanowires (AgNWs) via a simple water-bath pulling method to align the AgNWs embedded on polydimethylsiloxane (PDMS). Compared with sensors prepared by direct drop-casting or transfer-printing methods, our developed sensor represents a considerable breakthrough in both sensitivity and transparency. The maximum transmittance was 86.3% at a wavelength of 550 nm, and the maximum gauge factor was as high as 84.6 at a strain of 30%. This remarkably sensitive and transparent flexible sensor has strictly stable and reliable responses to motion capture and human body signals; it is also expected to be able to help monitor disabled physical conditions or assist medical therapy while ensuring privacy protection.

## Introduction

Highly stretchable and sensitive strain sensors are widely used in health monitoring, medical assistance and electronic skin applications because of their signal transmission functions^1–9^. To improve the sensor properties, two aspects are typically considered: material selection and sensor structure^10–13^, which means that either composite materials, such as graphene or carbon nanotubes (CNTs), are applied as the conductive layer to improve their capability^14–19^, or the entire sensor is designed to have a particular structure, such as an elastic porous sponge^20^. Although the most conductive and flexible materials are graphene and CNTs, they have a deep color, leading to low transparency^21^. Over the last few years, nanoparticles or mixtures of nanowires (NWs) and nanoparticles have been investigated^22–24^. Disappointingly, nanoparticles require high-temperature sintering to obtain sufficient conductivity^25,26^, resulting in the entire process being very complex and unacceptable for heat-sensitive materials.

In recent years, silver (Ag) NWs have been widely developed because of their outstanding mechanical and electrical properties^27–31^. Ha *et al*. used AgNWs as a sensing film and proposed multidimensional resistive strain sensors with a large gauge factor (GF) of >20 and a wide strain-detectable range of up to 60%^32^ .Polydimethylsiloxane (PDMS) is a common material in flexible sensors with excellent elasticity and transparency, and most proposed sensors are composed of AgNWs coated onto PDMS as the conductive layer, and the inactivity of PDMS ensures no reactivity with AgNWs. A sandwich-structured sensor of PDMS/AgNW/PDMS has been proposed^33^, which maintains complete encapsulation and avoids large-area irreversible deformation and translocation of AgNWs in the PDMS matrix. Because of the hydrophobicity of PDMS, more AgNW solution must be dropped onto PDMS to ensure good conductivity^34^. If the transfer-printing method is adopted^35^, loss of the AgNWs easily occurs during the transfer process. Neither the direct drop-casting nor the transfer-printing method can guarantee a highly uniform AgNW film, and both methods result in low sensitivity and transparency (maximum gauge factor is approximately 14^33^ and the sensor is nearly opaque).

Uniform and thin conductive films are vital to both the sensitivity and transparency of a sensor^36,37^. Ordered array structures are competitive for simultaneously maximizing the above two key features. Compared with patterning AgNWs on glass or PET^38,39^, there are more difficulties in forming ordered array structures on PDMS because of its hydrophobicity and large surface roughness and its flexible and elastic properties. These characteristics of PDMS limit the implementation of conventional aligning methods on it. In general, previous reports have sacrificed high sensitivity and transparency^40–42^, which are important for some situation. For example, in some applications, sensors are used to assist therapy or motion, such as heart rate monitors for heart disease patients, who are not willing monitors to tell tales of their health, so the transparency of sensors prevents monitors from privacy disclosure. Thus, balancing conductivity, sensitivity, transparency, tensile properties and reliability is particularly challenging for wearable and flexible electronics.

Here, we propose an effective and easy processing method by convection-induced interfacial self-assembly based on water-bath pulling, which is more easily available and cost-effective than some other methods, involving lithography technology, to orderly align AgNWs on PDMS^38^. For the first time, we fabricated two layers of AgNWs with uniform and regular orientation embedded on PDMS. Then, they were molded into a PDMS/two ordered AgNW array layers/PDMS sandwich structure to enhance the stability and reliability of the sensitive and transparent sensor. The prototype sensor with highly aligned networks of AgNWs fully satisfies the practical requirements of simultaneous high sensitivity, transparency and flexibility. This sensor has powerful potential to be applied for assisting motion detection, health monitoring and treatment, while the transparency of the sensor simultaneously protects the privacy of disabled people.

## Results and Discussion

### Ordered array structure

#### Stable floating interface

**Figure 1:**
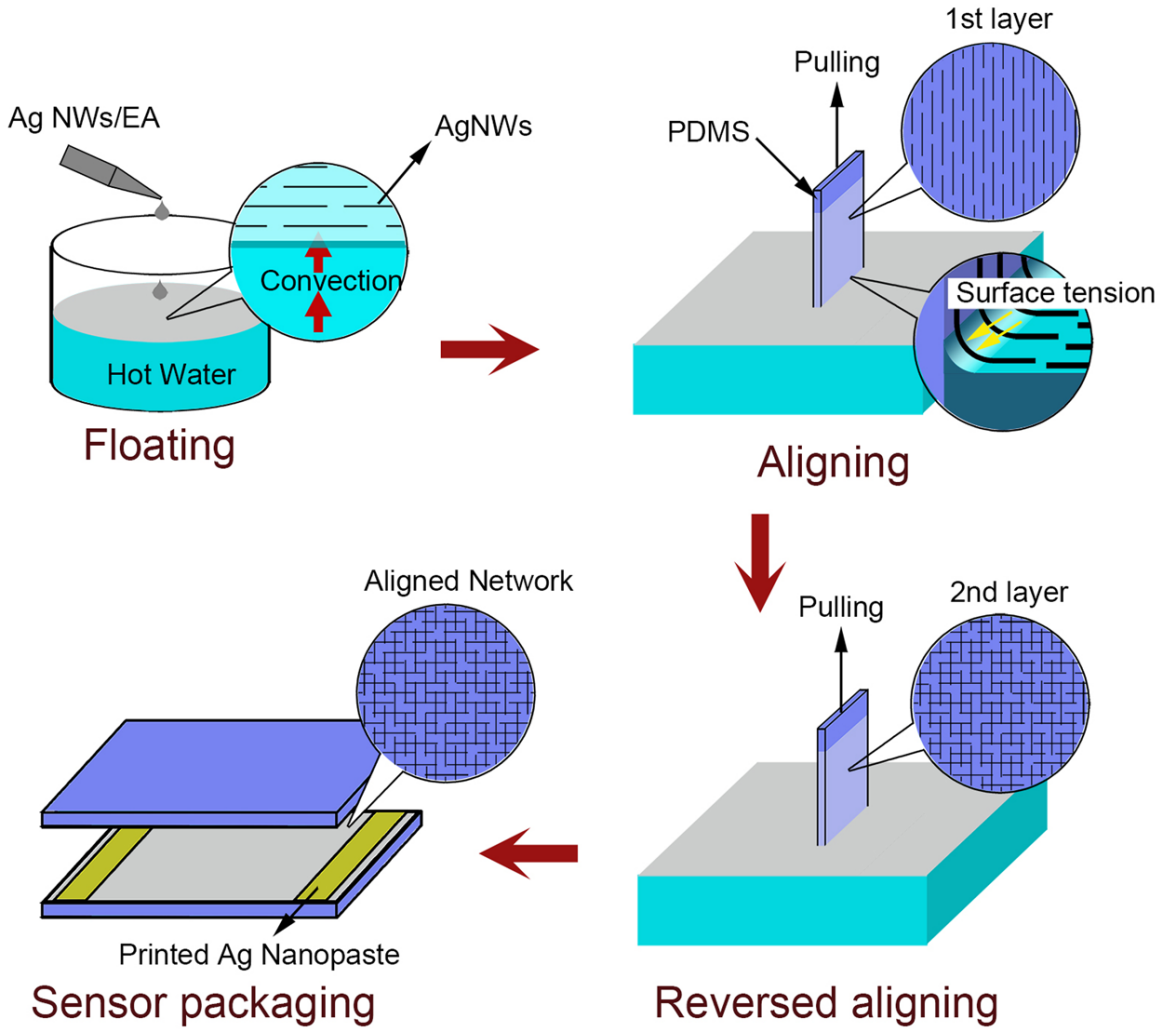
Fabrication process of the sensitive and transparent flexible sensor by the water-bath pulling method.

To obtain an ordered array structure of AgNWs on a flexible substrate, we propose a water-bath pulling method by pulling the substrate out of solution and aligning the AgNWs through convection-induced interfacial self-assembly. Generally, when dropping a droplet of a NW-ethanol solution into hot water, it will temporarily stay on the water surface instead of precipitating due to gravity and then spread because of the Marangoni effect, forming an ethanol/water interface. Moreover, the NWs will be directionally arranged in this thin interface by the shear force derived from the movement of two fluids against each other. By pulling the substrate out of solution, we successfully transferred NWs onto PDMS with order arrays. The detailed process is schematically shown in Fig. 1.

In previous reports^38,39,43^, upon continuously heating the bottom of a beaker, convection flow will form in the water, and upward-flowing and downward-flowing streams will appear in the center of and around the edge of the water in the beaker, respectively, which keep a droplet afloat on the surface of the water. To test that claim, we dropped the solution at the edge of the beaker where downward-flowing streams should exist and turned off the heating source to prevent water from sustaining a convection flow, and the droplet was supposed to precipitate rapidly in both situations. However, we still observed a thin layer of AgNWs floating on the water surface, which illustrates that the convection flow generated from heating is not the main reason for the formation of a stable interface (Fig. S1a).

A series of experiments was designed to explore the reason for this phenomenon, as shown in Fig. S1b–h. When the room-temperature AgNW solution was added to hot water, the contact interface instantly cooled, leading to a temperature difference between the solution surface and its bottom. Convection flow, referred to as an upward-flowing stream here, together with surface tension, helped the droplet overcome gravity. Regardless of the position of the droplet on the water surface, the upward-flowing stream would result in a floating state (Fig. S1f). If the AgNW solution temperature was higher than that of the water, convection flow from the surface to bottom (downward-flowing stream) occurred, which promoted precipitation (Fig. S1h). If the temperatures of the two solutions were approximately equal, no convection flow occurred, and the droplet quickly spread (Fig. S1d). Based on these observations, we speculated that the formation of a stable interface is probably due to local heat convection between the hot water and the AgNW solution (droplet of ethanol).

#### Aligning AgNWs on PDMS by pulling

To form a better and more stable ethanol/water interface, we increased the temperature of the deionized water to enhance the strong convection flow. In addition, we increased the concentration of the AgNW solution to ensure a closer arrangement of AgNWs at the interface. Furthermore, a lifting machine was utilized to provide a uniform and steady force to pull the PDMS substrate out of solution.

**Figure 2:**
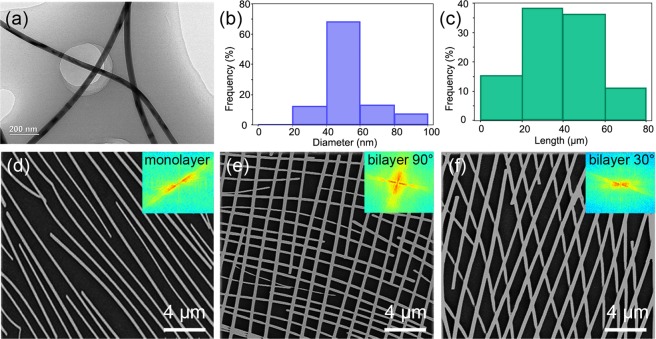
Characterization and ordered array structures of the AgNWs. (**a**) TEM images of AgNWs synthesized by a one-step polyol method. (**b**) The histogram of the diameters of the AgNWs. (**c**) The histogram of the lengths of the AgNWs. (**d**) SEM images of a single layer of AgNWs aligned by water-bath pulling. Inset: FFT analysis image. (**e**) SEM images of double layers of AgNWs with an angle of 90° aligned by water-bath pulling. Inset: FFT analysis image. (**f**) SEM images of double layers of AgNWs with an angle of 45° aligned by water-bath pulling. Inset: FFT analysis image.

Figure 2a–c show the appearance and characterization of the AgNWs after the one-step synthesis (see the experimental section for details). The diameter of the NWs was approximately 40–60 nm, and the dimensions of the wires were very uniform. Importantly, if the AgNWs were thinner and longer (larger length-to-diameter ratio), the ohmic contacts among the Ag wires formed more easily. However, wires that are too long may result in a disturbance and increase the difficulty of controlling their orientation during pulling. To gain a better directional orientation on PDMS, we utilized AgNWs with diameters of 40–60 nm and lengths of 30–50 μm (Fig. S2).

**Figure 3:**
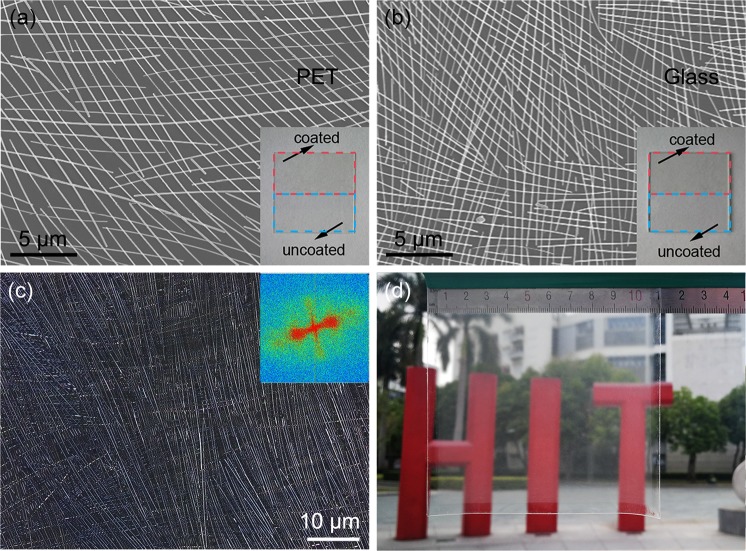
Adaptability of the water-bath pulling method on different substrates. (**a**) SEM images of double layers of aligned AgNWs coated on a PET substrate. Inset: contrast between the coated and uncoated substrate. (**b**) SEM images of double layers of aligned AgNWs coated on glass substrate. Inset: contrast between the coated and uncoated substrate. (**c**) CLSM image of double layers of aligned AgNWs on a large PDMS substrate. Inset: FFT analysis. (**d**) Optical image of double layers of aligned AgNWs on a large (10 cm × 10 cm) PDMS substrate. Its transparency is clearly outstanding since our university logo is clearly visible under real capturing conditions.

Compared with the NW alignments formed with traditional technologies, the orientation effect produced in this paper was more obvious in both the single and double layers (Fig. 2d–f). Fast Fourier transform (FFT) analysis by MATLAB, shown in the insets, indicated the presence of a very small radial angle in the single layer and nearly 90° and 30° in the double layers, suggesting that the AgNWs had a strong one-dimensional, controllable orientation on PDMS. Some other kinds of substrates, such as PET and glass, were tested using our method, and well-ordered array structures of AgNWs were also obtained (Fig. 3a,b). More importantly, through our optimized water-bath pulling method, these NWs can be aligned over a very large area with a multilevel orientation (Fig. 3c), and even on a substrate area of 10 cm × 10 cm, an orthogonal orientation of the AgNWs was successfully achieved (Fig. 3d). According to the confocal laser scanning microscope (CLSM) image and its FFT analysis, as shown in Fig. 3c, the two directional textures were very strong, and the radial angle was approximately 90°. According to our experiments, AgNWs were easily redissolved upon the second pulling and were rarely aligned in an obvious double-layered network structure. Mostly within a small area, some amount of ordering alignment for the double layer was present. To solve this problem, we dried the substrate in an oven at 70 °C for 5 min after the first pulling process. On the one hand, this process helps evaporate water and ethanol from the PDMS surface, maintaining a dry PDMS surface and makes preparations for the following pulling process. On the other hand, the adhesion between the AgNWs and PDMS substrate will be stronger to avoid dissolution in the next pulling operation. More experiments were designed to qualitatively control the space between adjacent AgNWs by water-bath pulling, and we found that higher temperatures of deionized water and higher concentrations and volumes of the/ethanol solution contribute to the close arrangement of AgNWs on the substrate, as shown in Fig. S3. More details about the experiments are discussed in the Supporting Information.

### Transparency and conductivity

These conductive ordered array structures prepared by our water-bath pulling method have many advantages in potential applications due to their excellent orientations. First, transparent conducting films are widely applied to numerous areas, such as solar cells and touch panels, in which high transparency and conductivity are needed. However, the color depth of conductive AgNW layers depends on the layer thickness and the NW arrangement. Stacking and overlapping of NWs are commonly applied in traditional methods to improve the conductivity at the expense of transparency. In addition, the PDMS substrate itself is strongly hydrophobic, which obstructs normal coating with a solution. To obtain better conductivity, the PDMS surface is typically coated with a thick layer of randomly aligned NWs by a direct drop-casting technique^44,45^. Amjadi *et al*. used spin coating to drop a solution onto glass^46^ and then transferred NWs to PDMS by peeling off the partially cured PDMS and avoiding the uneven distribution of AgNWs on PDMS. However, dropping a solution onto glass or a wafer cannot guarantee a uniform distribution of NWs, and spin coating has strict requirements for the concentration of the NW solution to control the film thickness. Furthermore, material consumption, including NWs falling off the substrate, leading to nonuniformity and open circuits in local areas, is also a notorious issue.

When the conductive layer is prepared by our water-bath pulling method, the AgNWs are attached to the PDMS surface by action of the upward force, and the water and ethanol will fall off the substrate because of the hydrophobicity of the substrate. After a 90° rotation of the above PDMS, an overlay layer of AgNWs was aligned on top of the previous layer to form an orthogonal array structure to enhance the ohmic contact to improve its conductivity. According to a four-point probe resistivity measurement system, its square resistance was just 25 Ω/sq. Due to the organized distribution of the AgNWs, minimal stacking and overlapping among wires occurred, which avoids redundant contacts that lead to low sensitivity and keeps the obtained conductive layer within a thin and uniform film. In addition, the NWs formed a regular mesh or grid, leading to a sensor with better transparency. After packaging with covered PDMS, the maximum transmittance of our strain sensors reached 86.3% with a resistance of only 168 Ω (because our sensors were sandwich structures of PDMS/AgNW/PDMS and their dimensions were kept constant, we characterize their conductivity by using resistance in the following section). However, for the sensor manufactured by transfer printing under the same specifications, the transmittance was less than 75% at the same resistance. If we pursue better transparency, excessive resistance will lead to poor performance.

**Figure 4:**
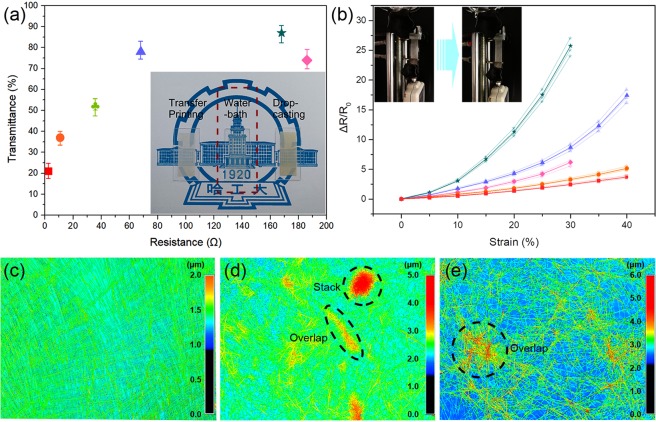
Optical and electrical performances of sensors fabricated by different methods: ■2.4 Ω by drop casting, •10.8 Ω by transfer printing, ♣36 Ω by transfer printing, ♦186 Ω by transfer printing, ▲68 Ω by water-bath pulling, ★168 Ω by water-bath pulling. (**a**) The transmittance of sensors as a function of resistance. Inset: optical image of three different sensors fabricated by transfer printing, water-bath pulling and drop casting. (**b**) ΔR/R_0_ versus tensile strain for various sensors. Height profiles of the AgNW network by (**c**) water-bath pulling, (**d**) drop casting and (**e**) transfer printing.

To increase the reliability of the results, we fabricated multiple sensors by each method and screened out three sensors which are close to each other in resistance value in each group, then we tested the transparency and sensitivity. Figure 4a shows the transmittance of the strain sensors under different resistances made with three methods. The results clearly indicate that the sensors made by the water-bath pulling method have a better transmittance, exceeding 80%. In contrast, ultralow transparency was present after drop casting, and the transmittance was only 21%. For the sensors obtained by the transfer method, the transmittance was below 80%. The appearance of the usable strain sensors fabricated by the three methods is shown in the inset of Fig. 4a, including sensors fabricated by the transfer-printing, water-bath pulling and direct drop-casting methods. Obviously, the middle sensor has the best transparency among the three samples. We can clearly observe the numbers, characters and patterns of our university logo. In contrast, the other two sensors possess poor transparency because of the thick AgNW layer and their random distribution. According to the experiments above, we can conclude that a large thickness of the AgNW layer contributes more to conductivity but reduces the transmittance of the sensors.

Figure 4c–e show the height profiles of the AgNW networks fabricated by the three methods. In Fig. 4c, AgNWs are distributed uniformly, and a regular grid structure can be obviously seen. The height is distributed mainly within the range of 1.0–1.5 μm. In Fig. 4d, more AgNWs were observed and distributed irregularly, and stacking and overlapping can be found, resulting in a wider range of height distribution, mainly from 1.5 to 5.0 μm. In Fig. 4e, few stacks were found because AgNWs at the bottom of a stack on the substrate cannot be transferred due to not touching the liquid PDMS, but overlapping was still observed. The height distribution was mainly from 2.5 to 6.0 μm. More AgNWs were lost during transfer, resulting in a sparse network.

### Sensitivity and repeatability

Next, we tested the mechanical properties and sensitivity of the sandwich strain sensor with an ordered array structure of AgNWs. The test results of average value are shown in Fig. 4b. Three original ΔR/R_0_-Strain curves were depicted with similar lighter color in each group.

During the stretching process, if the stretching amplitude was too large, the resistance change went beyond the measurement range. At this point, we believe that the sensor reached the maximum stretch. We found that the conductive layer in the sensor had a remarkable effect on its sensitivity and stretchability. The conductive layer formed using direct drop-casting possessed a very small resistance (only 2.4 Ω); however, little resistance change occurred during stretching^46^, which indicated that the sensitivity of the entire sensor was very low, regardless of micro- or large-scale stretching. For the sensor formed by the transfer method, conductive layers with different resistances were obtained by controlling the solution concentration (amount of AgNWs). When an application requires very good conductivity, a sensor can be fabricated with a small resistance (10.8 Ω) using a high solution concentration. Similar to the above discussion, the tensile performance was good, but the sensitivity was poor. By increasing the resistance using a low solution concentration, the gauge factor (GF) during large-scale stretching was considerably increased and reached a maximum of 19.6 when the resistance was 186 Ω. However, unfortunately, the stretching ability was still poor, with a maximum strain of 30%. Then, we compared two sensors formed using the water-bath pulling method with resistances of 168 Ω and 68 Ω. Surprisingly, we found that these sensors were much more sensitive than the sensors made by the other two methods. In particular, during small-scale stretching, these sensors maintained a relatively high sensitivity (GF is 29.3 and 16.7 at a strain of 10%, respectively), which has rarely been reported before. Furthermore, the maximum GF of the sensor with a 168 Ω resistance during large-scale stretching reached 84.6, far greater than the values reported in the literature^35,46^. To gain stable tensile properties, we further increased the number of droplets in the water-bath pulling method to obtain a sensor with a resistance of 68 Ω, and its largest GF was approximately 43 with a good stretching performance when the strain was 40%.

To conclude, the reason that the sensitivity of our sensor is better than that of those made by drop-casting and transfer-printing methods is that our sensor with an aligned AgNW structure is more uniform than the AgNW conductivity layer formed by other methods, avoiding stacking and overlapping dramatically, which prevent AgNWs from moving with the PDMS when strain is applied. According to simulations, when only considering unidirectional NWs that are stretched parallel to the alignment direction, the junctions quickly disconnect, resulting in sparse NWs^46^. Relatively, the NWs squeezed in the vertical direction are more closely arranged. However, considering the above two factors, if the AgNWs are aligned in an orthogonal arrangement, changing the stretching direction will result in a large resistance change (high sensitivity). Meanwhile, the squeezed wires (vertical direction) will be more closely arranged, resulting in more probable connections with wires in the orthogonal direction to ensure a conductive pathway. Due to these reasons, the proposed sensor with an ordered array structure maintains both a high sensitivity and good tensile properties^47^. Additionally, the thinness of the conductive layer contributes greatly to the sensor sensitivity. The conductive layer is much thinner in our sensor made by water-bath pulling (only two AgNW layers) than that in sensors made by the drop-casting (more AgNW solution is applied due to the hydrophobicity of PDMS) and transfer-printing (to compensate for the loss of AgNWs, the conductive layer is much thicker) methods, as shown in the inset of Fig. 4a.

For NW-material sensors, during stretching, the NWs will sustain excessive displacement, which may cause the entire sensor to fail due to open conductive paths. In this way, if the recovery capability of the sensor is poor or if the NWs sustain large and permanent displacement, the entire sensor will lose function or fail to work. Our sandwich-structure sensors with an ordered array structure of AgNWs have natural advantages in terms of reliability. The package structure avoids the entry of external impurities and humidity and the loss of AgNWs. In addition, the complete encapsulation ensures that the AgNWs quickly recover to their original position with the recovery of the PDMS after stretching, which guarantees that the sensors function repeatedly and reliably.

### Sensing and application of the strain sensor

**Figure 5:**
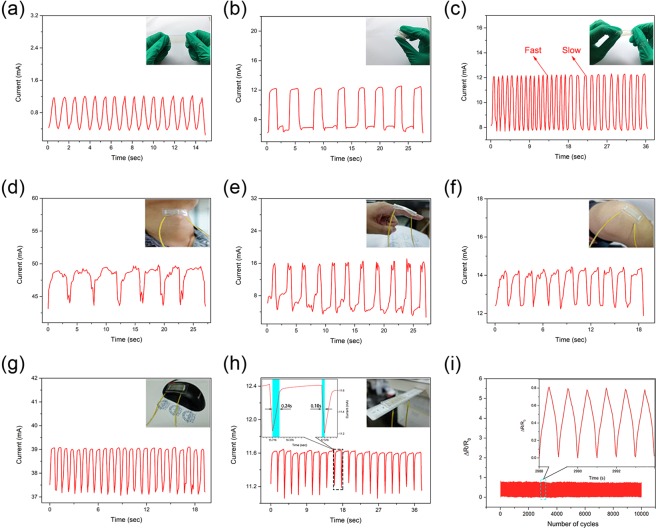
Repeatability testing and applications. (**a**) Stretching test. (**b**) Bending test. (**c**) Twisting test. (**d**) Swallowing detection. (**e**) Finger bending detection. (**f**) Knee movement detection. (**g**) Mouse clicking detection. (**h**) Ruler twisting detection, inset: Response time of ruler twisting detection. (**i**) Repeatability test.

It is noticeable that upon stretching the sensor, despite the protection from encapsulation, the NWs will be stressed by the deformation of PDMS, which is one reason for the resistance change in a sensor. In general, a sensor demonstrates good recovery after microstretching. However, when a sensor undergoes larger stretching movements, such as finger bending or knee moving, the AgNWs will gather, resulting in NWs missing from some areas, which may cause an open circuit. In our sensor fabricated by water-bath pulling, the mentioned problems can be solved. Figure 5 shows the signal monitoring of our strain sensors during stretching (Fig. 5a), bending (Fig. 5b), and twisting (Fig. 5c). Repeated and periodic peaks indicate quick and strict responses when the sensor is loaded with regular or irregular stress inputs. As an example, in Fig. 5b, the different shapes of the signal curve correspond to a series of deformations (displacements) of the sensor. The sudden appearance of peaks and troughs suggests resistance changes during the stress cycles. The rapid decreases and increases in the signal indicate that the sensor has a rather good response time (which is related to the speed of the movement), amplitude and repeatability. Moreover, the similar shape of each cycle indicates the satisfactory recovery performance of the strain sensor over repeated cycles. Thus, the sandwich strain sensor with an ordered array structure of AgNWs has noticeable advantages in both performance and reliability, in addition to good transparency, conductivity, and sensitivity.

Figure 5d–f show the high sensitivity of the sensor for health monitoring. The current-time curves indicate that changes in the current signals over time were strictly modulated by human behaviors, including throat swallowing (Fig. 5d), finger bending (Fig. 5e) and knee movement (Fig. 5f). Interestingly, when we performed these operations, signal interruption phenomena did not appear, which indicated the good tensile properties of the sensor with an ordered array structure of AgNWs under large strain. Various articular or muscular movements produced unique curve shapes due to the completely different deformations of the sensor. Additionally, each curve possessed repeated shapes accompanying regular movements, proving that the sensor has superior recoverability and a potential application in motion recognition.

Commonly, reported strain sensors responding to mechanical deformation by a resistance change have low sensitivity, resulting in small signal changes when slightly stretched (Fig. 4b shows that an ordinary resistance sensor displays a small change when the strain (ε) is less than 10%). However, our sensors with an ordered array of AgNWs were able to detect a mild touch. The signal curves derived from microstretching, such as clicking a mouse (Fig. 5g) and mildly twisting the ruler (Fig. 5h), in which the minimum response time was as low as 0.1 s (Fig. 5h inset), were investigated. When touched, the sensor responded very quickly and displayed a large signal change. The durability of the sensor was detected by bending for more than 10,000 cycles, as shown in Fig. 5i, according to which we can conclude that the stability of the sensor is fairly good. Above all, our sensor can monitor different motions with a corresponding feature signal curve, which can be discriminated by its shape. The sensor performed well in stability with sharp generated current decreases and increases, which possessed obvious regularity, and rapid speed of response and recovery.

**Figure 6:**
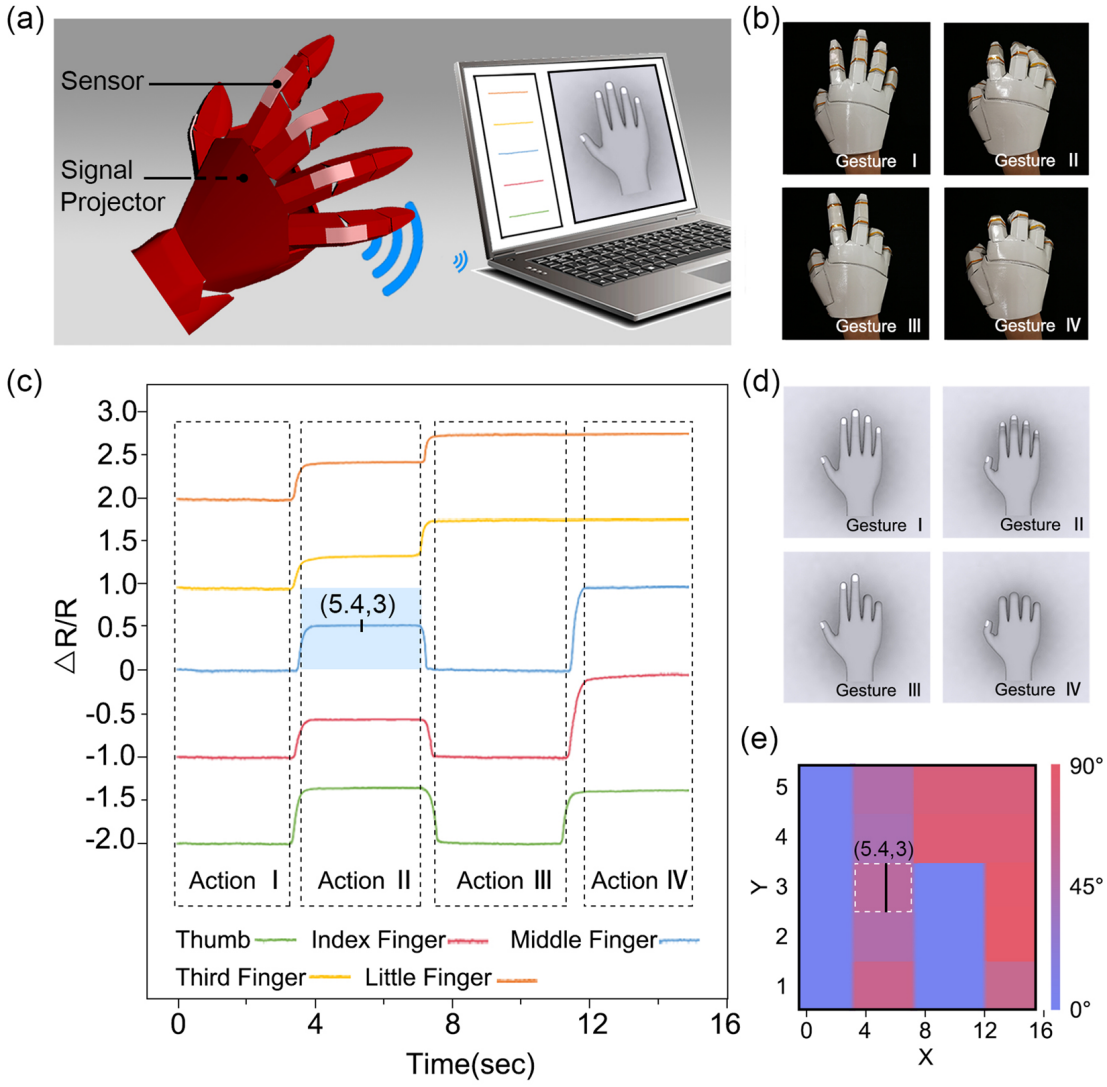
Application of the real-time finger gesture recreation. (**a**) Schematic of the sample structure and working process. (**b**) Optical image of a series of different finger gestures. (**c**) ΔR/R_0_ versus time for various sensors on the glove due to serial finger gestures. (**d**) Simulated image recreation of the finger gestures corresponding to (**c**) based on the motion of (**b**). (**e**) The corresponding bending angle mapping according to (**c**).

Finally, five stretchable and transparent sensors were assembled into a glove, and a real-time gesture detector was obtained, as shown in Fig. 6a. Generally, to completely recreate finger movements, fourteen sensors should be applied for each hand since each hand has fourteen joints. However, here, we only used five sensors to simplify the model. These sensors were attached to finger joints on the glove surface, and bending or stretching was achieved with fingers in the glove. Figure 6a schematically shows the working mode of the finger gestures and their motion recreation in the virtual computer. For every faint movement of the fingers in the glove, the monitored signal of the relevant sensors varied. The monitored data were then transferred wirelessly to a computer and exhibited as curves of ΔR/R-time in a customized program. The degree of the monitored signal variation reflected how much the finger bent, and the gesture could be recreated accurately and in real time. A series of finger movements with our glove are shown in Fig. 6b. Data received from the five sensors were acquired simultaneously and then processed by the self-designed software into the corresponding curves, as shown in Fig. 6c. Platforms in the curve indicate that the sensor was at rest (no movement), and the up- and down-hill changes indicated bending and extending of the fingers, respectively. The bending angle was calculated according to the relative height of the platform to the original for the sensitivity and stability of sensors; thus, for example, the state of the middle finger in Fig. 6c at 5.4 s can be recorded by using the time-bending angle planar reference frame as noted (5.4, 3) in Fig. 6e. Finally, the corresponding finger gestures were recreated precisely according to the obtained bending angles, as shown in Fig. 6d.

Such a highly sensitive and transparent sensor can also be adapted to help the disabled achieve dynamic health monitoring and medical treatment. For example, our sensor can be applied to analyze health situations combined with big data. Additionally, the high transparency of the sensor allows patients to discretely wear the sensor and avoid discrimination, which enables patients to receive physical therapy while preventing secondary damage from psychological sensitivity. Based on this transparent, stretchable, sensitive, reliable and multifunctional sensor, the realization of flexible sensors integrated with the human body is expected to enter the big data era.

## Conclusions

In summary, we reported an aligned AgNW network structure as a conductive layer on PDMS by a water-bath pulling method in a flexible and wearable strain sensor to provide better sensitivity, transmittance and stability performance. The fabrication process was very simple, but the effect was outstanding due to the peculiar orthogonal orientation and sandwich-structure encapsulation. The sensor possessed ultrahigh sensitivity with a large gauge factor of 84.6 at a strain of 30%. Furthermore, the thickness of the AgNW film was controlled by the concentration of droplets during the pulling process, and ultratransparency was achieved. Finally, the sensor was applied to the detection of human muscle and touching motions and demonstrated excellent reliability and repeatability, fast response and high stability. Thus, combined with its ultrahigh transparency, the sensor can be applied in motion detection and health monitoring, particularly for disabled people to fully protect their privacy. Based on the ability of monitoring data to construct a large database, we can conduct remote monitoring and control to provide the timeliest and best treatment plans.

## Methods

### Preparation of AgNWs

The AgNWs were synthesized via a one-step polyol method^48–51^. AgNO_3_ (99.8%, AR) and glycerol were used as the reactants. Polyvinylpyrrolidone (PVP) with Mw = 1,300,000 g/mol and Mw = 650,000 g/mol were mixed as coating agents with a molar ratio of 2:1. AgNO_3_ was dissolved in glycerol (15 ml) and stirred at room temperature by magnetic stirring with a stirring speed of 60 rpm. The mixed PVP was dissolved in glycerol (20 ml) and stirred under a heating temperature of 65 °C with a stirring speed of 260 rpm. After complete stirring, NaCl (150 μM, 2 ml) was added to the PVP solution, and the resulting solution was stirred at a constant temperature of 50 °C. Next, the AgNO_3_ solution was dropped into the mixed solution and stored for 5 min, and then, the solution was poured into the reaction vessel to react at 155 °C for 4 h. After the reaction, the solution was washed with ethanol at a volume ratio of 1:3 and centrifuged three times at 4000 rpm for 10 min to remove the excess PVP and impurities. Finally, the AgNWs were stored in ethanol for further experiments, and AgNW/EA (AgNWs in ethanol) with a mass fraction of 0.4% was obtained. The average diameter and length of the AgNWs were approximately 40–60 nm and 30–50 μm, respectively.

### Fabrication of the sensor with an ordered array structure

The fabrication process of the sensor is schematically shown in Fig. 1. The PDMS substrates were first prepared by a mask (5.5 cm × 2.5 cm with a thickness of 0.5 mm). The beaker was filled with deionized water and heated to a certain temperature on a heating plate and placed under the chuck. When these steps were complete, the previously prepared AgNW solution was dropped into the beaker by a pipette, and a thin layer of AgNWs will float on the surface of water instead of depositing. Next, the chuck and PDMS were immersed into the deionized water and then lifted at a speed of 20 mm/min. The thin layer of AgNWs will be absorbed onto the surface of PDMS under the effect of Van der Waals force. When the pulling process was finished, the AgNWs had been transferred onto PDMS from the water surface. After the first water-bath pulling step, the PDMS was heated in an oven for 5 min. Then, the above procedure was repeated after rotating the PDMS substrate by 90°. Finally, a substrate with a double-layered AgNW network array was obtained.

The excess AgNWs were removed using tape at the edge of PDMS. To monitor the electrical signals, two electrodes printed with Ag nanoparticle paste were obtained by an ink-jet printer (DM02831, FUJI) on both sides of the aligned AgNW conductive layer. Finally, the sensor was encapsulated with liquid PDMS and cured at 80 °C for 1 h to ensure its stability and reliability.

### Characterization

The microstructures of the AgNWs were characterized by a scanning electron microscope (SEM, HITACHI S-4700), and more subtle structures were observed by a transmission electron microscope (TEM, Tecnai G2 Spirit 120 kV). The transmittance was measured by UV-visible spectrophotometry (UV-2600). The tensile properties were measured by a digital tensile machine (HANDPI HPB). The current changes were measured dynamically using an electrochemical workstation (CHI760D). The morphology of the oriented AgNWs on a large-area substrate was captured using CLSM (VK-X200).

## Supplementary information

Supplementary Information

## References

[1] Amjadi M, Kyung KU, Park I, Sitti M (2016). Stretchable, Skin-Mountable, and Wearable Strain Sensors and Their Potential Applications: A Review. Advanced Functional Materials.

[2] Shuai XT (2017). Highly Sensitive Flexible Pressure Sensor Based on Silver Nanowires-Embedded Polydimethylsiloxane Electrode with Microarray Structure. ACS Applied Materials & Interfaces.

[3] You B, Han CJ, Kim Y, Ju BK, Kim JW (2016). A Wearable Piezocapacitive Pressure Sensor with a Single Layer of Silver Nanowire-based Elastomeric Composite Electrodes. Journal of Materials Chemistry A.

[4] Yao, S. S. *et al*. Silver Nanowire Strain Sensors for Wearable Body Motion Tracking. *IEEE Sensor*, 1823–1826 (2015).

[5] Wang LL (2017). High-performance, Flexible Electronic Skin Sensor Incorporating Natural Microcapsule Actuators. Nano Energy.

[6] Choi S, Lee H, Ghaffari R, Hyeon T, Kim DH (2016). Recent Advances in Flexible and Stretchable Bio-Electronic Devices Integrated with Nanomaterials. Advanced Materials.

[7] Trung TQ, Duy LT, Ramasundaram S, Lee NE (2017). Transparent, Stretchable, and Rapid-response Humidity Sensor for Body-attachable Wearable Electronics. Nano Research.

[8] Nag A, Mukhopadhyay SC, Kosel J (2017). Wearable Flexible Sensors: A Review. IEEE Sensors Journal.

[9] Ma, Y. J. *et al*. Soft Elastomers with Ionic Liquid-Filled Cavities as Strain Isolating Substrates for Wearable Electronics. *Small* **13**, 10.1002/Smll.201602954 (2017). 10.1002/smll.201602954 PMC5332287 28026109

[10] Liao XQ (2015). Flexible and Highly Sensitive Strain Sensors Fabricated by Pencil Drawn for Wearable Monitor. Advanced Functional Materials.

[11] Bao RR (2015). Flexible and Controllable Piezo-Phototronic Pressure Mapping Sensor Matrix by ZnO NW/p-Polymer LED Array. Advanced Functional Materials.

[12] Boutry CM (2015). A Sensitive and Biodegradable Pressure Sensor Array for Cardiovascular Monitoring. Advanced Materials.

[13] Jian, M. Q. *et al*. Flexible and Highly Sensitive Pressure Sensors Based on Bionic Hierarchical Structures. *Advanced Functional Materials* **27**, 10.1002/Adfm.201606066 (2017).

[14] Tuteja, S. K., Ormsby, C. & Neethirajan, S. Noninvasive Label-Free Detection of Cortisol and Lactate Using Graphene Embedded Screen-Printed Electrode. *Nano-Micro Letters* **10**, 10.1007/s40820-018-0193-5 (2018). 10.1007/s40820-018-0193-5 PMC6199085 30393690

[15] Singh E, Meyyappan M, Nalwa HS (2017). Flexible Graphene-Based Wearable Gas and Chemical Sensors. ACS Applied Materials & Interfaces.

[16] Jeykumari DRS, Kalaivani R, Narayanan SS (2012). Nanobiocomposite Electrochemical Biosensor Utilizing Synergic Action of Neutral Red Functionalized Carbon Nanotubes. Nano-Micro Letters.

[17] Zhou J, Xu XZ, Yu H, Lubineau G (2017). Deformable and Wearable Carbon Nanotube Microwire-based Sensors for Ultrasensitive Monitoring of Strain, Pressure and Torsion. Nanoscale.

[18] Shi G (2016). Highly Sensitive, Wearable, Durable Strain Sensors and Stretchable Conductors Using Graphene/Silicon Rubber Composites. Advanced Functional Materials.

[19] Kim KH (2017). Wearable Resistive Pressure Sensor Based on Highly Flexible Carbon Composite Conductors with Irregular Surface Morphology. ACS Applied Materials & Interfaces.

[20] Zhang H (2016). Piezoresistive Sensor with High Elasticity Based on 3D Hybrid Network of Sponge@CNTs@Ag NPs. ACS Applied Materials & Interfaces.

[21] Xu MX, Qi JJ, Li F, Zhang Y (2018). Highly Stretchable Strain Sensors With Reduced Graphene Oxide Sensing Liquids for Wearable Electronics. Nanoscale.

[22] Azadbakht, A., Abbasi, A. R., Derikvand, Z., Karimi, Z. & Roushani, M. Surface-Renewable AgNPs/CNT/rGO Nanocomposites as Bifunctional ImpedimetricSensors. *Nano-Micro Letter*s **9**, 10.1007/S40820-016-0101-9 (2017). 10.1007/s40820-016-0101-9 PMC6223774 30460301

[23] Chen, Y. R., *et al*. Roller-Induced Bundling of Long Silver Nanowire Networks for Strong Interfacial Adhesion, Highly Flexible, Transparent Conductive Electrodes. *Scientific Reports* **7**, 10.1038/S41598-017-16843-Y (2017). 10.1038/s41598-017-16843-y PMC5709471 29192222

[24] Zhou, Q. *et al*. A Hierarchical Nanostructure-Based Surface-Enhanced Raman Scattering Sensor for Preconcentration and Detection of Antibiotic Pollutants. *Advanced Materials Technologies* **2**, 10.1002/Admt.201700028 (2017).

[25] Koga H (2016). A High-sensitivity Printed Antenna Prepared by Rapid Low-temperature Sintering of Silver Ink. RSC Advances.

[26] Markina M, Stozhko N, Krylov V, Vidrevich M, Brainina K (2017). Nanoparticle-based Paper Sensor for Thiols Evaluation in Human Skin. Talanta.

[27] Yoon SS, Khang DY (2016). Facile Patterning of Ag Nanowires Network by Micro-Contact Printing of Siloxane. ACS Applied Materials & Interfaces.

[28] Song WG (2016). Sensitive and Stretchable Strain Sensors Based on Silver Nanowires Network. Journal of Nanoscience and Nanotechology.

[29] Duan, S. S., *et al*. A Highly Stretchable, Sensitive, and Transparent Strain Sensor Based on Binary Hybrid Network Consisting of Hierarchical Multiscale Metal Nanowires. *Advanced Materials Technology* **3**, 10.1002/admt.201800020 (2018).

[30] Lai YC (2016). Extraordinarily Sensitive and Low-Voltage Operational Cloth-Based Electronic Skin for Wearable Sensing and Multifunctional Integration Uses: A Tactile-Induced Insulating-to-Conducting Transition. Advanced Functional Materials.

[31] Kim, J., Park, J., Jeong, U. & Park, J. W. Silver Nanowire Network Embedded in Polydimethylsiloxane as Stretchable, Transparent, and Conductive Substrates. *Journal of Applied Polymer Science* **133**, 10.1002/App.43830 (2016).

[32] Ha SH, Ha SH, Jeon MB, Cho JH, Kim JM (2018). Highly Sensitive and Selective Multidimensional Resistive Strain Sensors Based on a Stiffness-variant Stretchable Substrate. Nanoscale.

[33] Amjadi M, Pichitpajongkit A, Lee S, Ryu S, Park I (2014). Highly Stretchable and Sensitive Strain Sensor Based on Silver Nanowire-Elastomer Nanocomposite. ACS Nano.

[34] Choi TY (2017). Stretchable, Transparent, and Stretch-Unresponsive Capacitive Touch Sensor Array with Selectively Patterned Silver Nanowires/Reduced Graphene Oxide Electrodes. ACS Applied Materials & Interfaces.

[35] Liu HS, Pan BC, Liou GS (2017). Highly Transparent AgNW/PDMS Stretchable Electrodes for Elastomeric Electrochromic Devices. Nanoscale.

[36] Wang, Q., Jian, M. Q., Wang, C. Y. & Zhang, Y. Y. Carbonized Silk Nanofiber Membrane for Transparent and Sensitive Electronic Skin. *Advanced Functional Materials* **27**, 10.1002/Adfm.201605657 (2017).

[37] Lee D (2016). Highly Sensitive, Transparent, and Durable Pressure Sensors Based on Sea-Urchin Shaped Metal Nanoparticles. Advanced Materials.

[38] Duan SK (2015). Water-bath Assisted Convective Assembly of Aligned Silver Nanowire Films for Transparent Electrodes. Physical Chemistry Chemical Physics.

[39] Cho S (2017). Large-Area Cross-Aligned Silver Nanowire Electrodes for Flexible, Transparent, and Force-Sensitive Mechanochromic Touch Screens. ACS Nano.

[40] Joo Y (2015). Silver Nanowire-embedded PDMS with a Multiscale Structure for a Highly Sensitive and Robust Flexible Pressure Sensor. Nanoscale.

[41] Wang JX, Yan CY, Kang WB, Lee PS (2014). High-efficiency Transfer of Percolating Nanowire Films for Stretchable and Transparent Photodetectors. Nanoscale.

[42] Guo HY (2017). Transparent, Flexible, and Stretchable WS_2_ Based Humidity Sensors for Electronic Skin. Nanoscale.

[43] Ahn K, Kim D, Kim O, Nam J (2015). Analysis of Transparent Conductive Silver Nanowire Films From Dip Coating Flow. Journal of Coatings Technology and Research.

[44] Asadnia, M. *et al*. From Biological Cilia to Artificial Flow Sensors: Biomimetic Soft Polymer Nanosensors with High Sensing Performance. *Scientific Reports* **6**, 10.1038/Srep32955 (2016). 10.1038/srep32955 PMC5020657 27622466

[45] Costa THD, Choi JW (2017). A Flexible Two dimensional Force Sensor Using PDMS Nanocomposite. Microelectronic Engineering.

[46] Lee, S., Amjadi, M., Pugno, N., Park, I. & Ryu, S. Computational Analysis of Metallic Nanowire-elastomer Nanocomposite Based StrainSensors. *AIP Advance*s **5**, 10.1063/1.4936635 (2015).

[47] Yang M (2018). Facile and Highly Efficient Fabrication of Robust Ag Nanowire-elastomer Composite Electrodes with Tailored Electrical Properties. Journal of Materials Chemistry C..

[48] Ran YX, He WW, Wang K, Ji SL, Ye CH (2014). A One-step Route to Ag Nanowires with a Diameter Below 40 nm and an Aspect Ratio Above 1000. Chemical Communications.

[49] Hwang J, Shim Y, Yoon SM, Lee SH, Park SH (2016). Influence of Polyvinylpyrrolidone (PVP) Capping Layer on Silver Nanowire Networks: Theoretical and ExperimentalStudies. RSC Advances.

[50] Coskun S, Aksoy B, Unalan HE (2011). Polyol Synthesis of Silver Nanowires: An Extensive Parametric Study. Crystal Growth & Design.

[51] Wang, S., Tian, Y. H., Ding, S. & Wang, C. Q. The Role of Chloride Ions in Rapid Synthesis of Ultra-long Silver Nanowires for Flexible Electrodes. *Materials Research Express* **3**, 10.1088/2053-1591/3/7/075007 (2016).

