# Supplementary material for: Highly Sensitive and Transparent Strain Sensors with an Ordered Array Structure of AgNWs for Wearable Motion and Health Monitoring
Source: Sci Rep. 2019 Feb 20;9:2403. doi: 10.1038/s41598-019-38931-x (PMC6382792; doi:10.1038/s41598-019-38931-x)
Supplement: Supplementary file 1 — Supplementary Information [file 41598_2019_38931_MOESM1_ESM.pdf]

Supporting Information

Highly Sensitive and Transparent Strain Sensors with an Ordered Array Structure of AgNWs for Wearable Motion and Health Monitoring

Fanqi Yin[1,2,#], Huajun Lu[1,2,#], Hao Pan[1,2,#], Hongjun Ji[1,2,*], Shuai Pei[1,2], Hao Liu[1,2], Jiayi Huang[1,2], Jiahui Gu[1,2], Mingyu Li[1,2] and Jun Wei[3]

[1] State Key Laboratory of Advanced Welding and Joining, School of Materials Science and Engineering, Harbin Institute of Technology at Shenzhen, Shenzhen 518055, P. R. China

[2] Center of Flexible and Printable Electronics, Harbin Institute of Technology (Shenzhen), Shenzhen 518055, P. R. China

[3] Singapore Institute of Manufacturing Technology, 73 Nanyang Drive, 637662, Singapore

*Correspondence to jhj7005@hit.edu.cn

Dropping Experiments

According to Duan's theory, the up-stream is important for forming the ethanol/water interface because of heating source. However, **Figure S1a** proved that a floating state appeared when the AgNWs solution was dropped to the edge of the beaker where the downstream occurred. Furthermore, if removing heating source, as shown in **Figure S1e** and **S1f**, the ethanol/water interface also maintained. Importantly, if the temperature of AgNWs solution was higher, a direct precipitation replaced the former phenomenon and no interface existed, as shown in **Figure S1g** and **S1h**. In **Figure S1b** and **S1c**, the temperature was kept as the same for both the AgNWs solution and the water. The droplet diffused firstly and then dissolved in water (**Figure S1d**). Based on these designed experiments, we discovered that the temperature difference is probably the main cause of the above interesting phenomena.

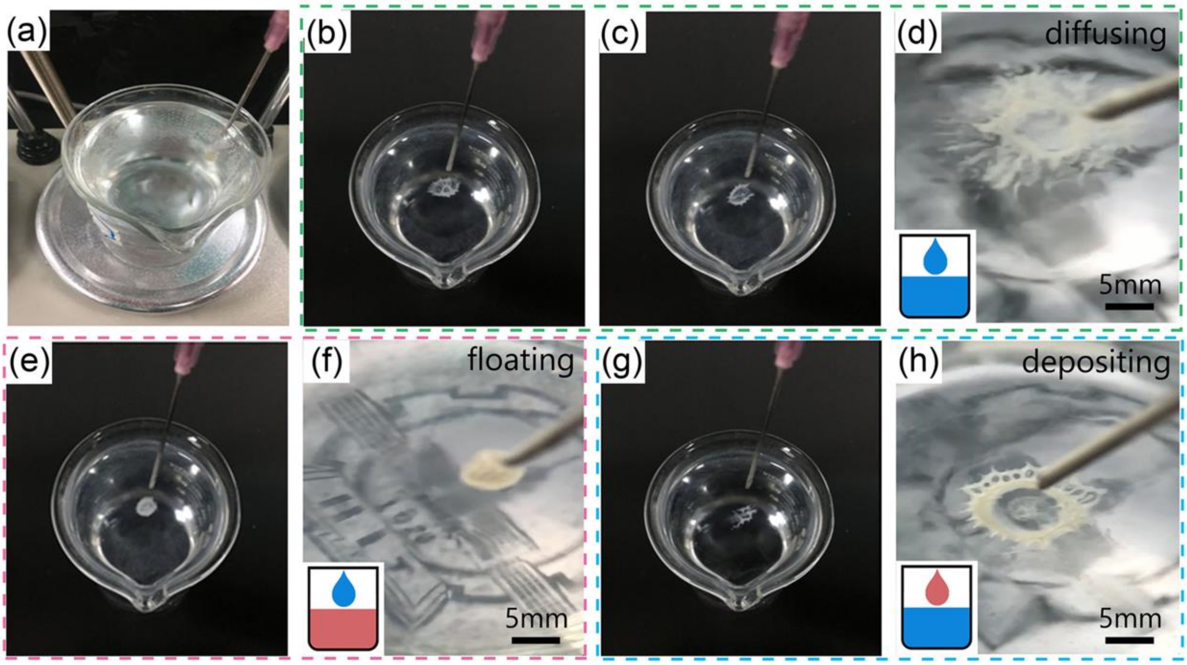

Figure S1. Optical images of a serial of drop experiments using (a) AgNWs/EA (RT, room temperature) dropped into water (70 ℃) with a heating source, (b) AgNWs/EA (RT) dropped into water (RT) without heating source, (c) AgNWs/EA (70 ℃) dropped into water (70 ℃), (d) Diffusing state of AgNWs, (e) AgNWs/EA (RT) dropped into water (70 ℃), (f) Floating

state of AgNWs, (g) AgNWs/ EA (70 ℃) dropped into water (RT) and (h) Depositing state of AgNWs.

Characterization of Synthesized Silver Wires

The morphology of the synthesized AgNWs is uniform, and no particulate Ag is found in the SEM images as seen in **Figure S2**. The length and diameter of AgNWs distribute mainly in the range between 30-50 μm and 40-60 nm, respectively, which is proved to be suitable for water-bath pulling.

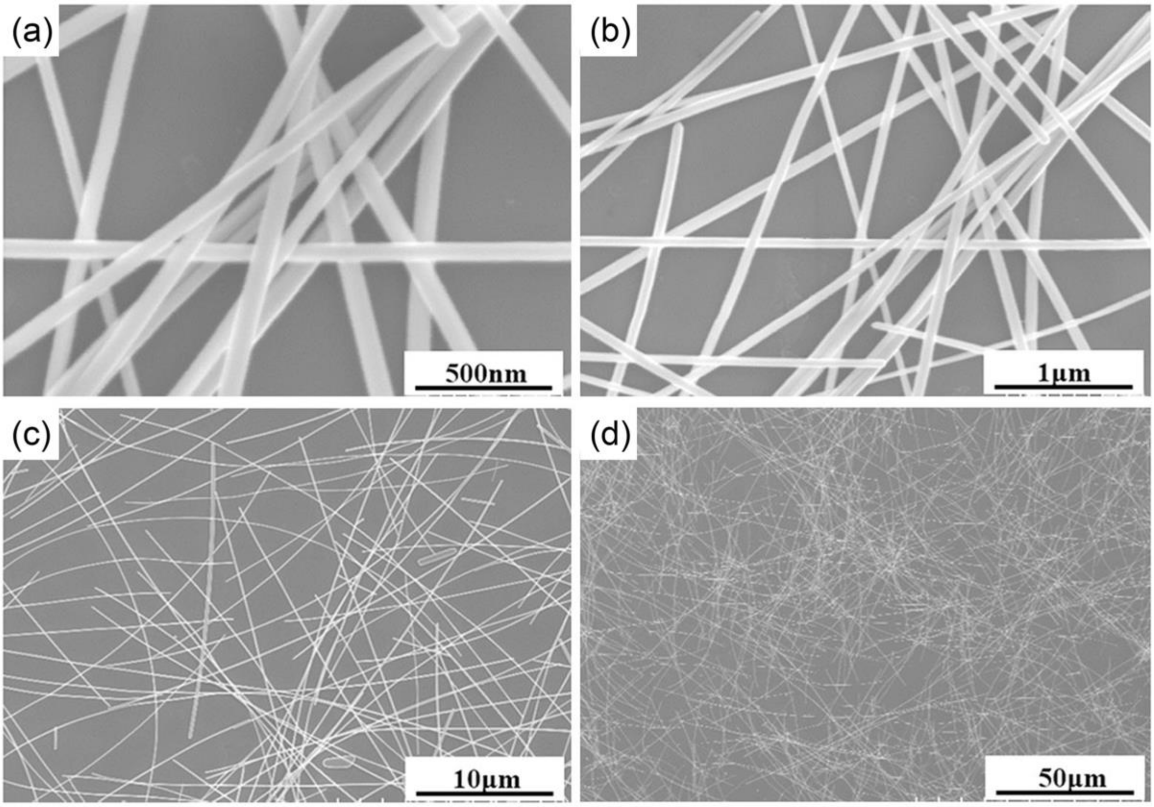

Figure S2. SEM images of synthesized AgNWs with different magnification.

Controlling the Interval between the Adjacent AgNWs

Many factors are investigated in water-bath pulling to explore their effects on the interval between the adjacent AgNWs. The following ones play the most important roles.

Water Temperature

0.3 ml solution with 0.4 wt.% AgNWs was dropped into deionized water when heated to a certain temperature. **Figure S3a** to **S3c** illustrate the distribution of AgNWs on the PDMS substrate when they aligned under 40 °C, 60 °C and 80 °C, respectively. To quantitatively characterize the interval accurately, three lines were drawn in each image, and the interval is calculated by the length of the line dividing the number of AgNWs. The average interval can be obtained (**Figure S3j**). With the increase of water temperature, the interval between the adjacent AgNWs is shorter. We speculate the reason is that higher temperature of the deionized water induces higher temperature gradient between the hot water and the AgNW solution, so that the stronger local heat convection contributes more AgNWs floating on the water-air surface.

AgNWs Concentration

Figure S3d to **S3f** show AgNWs array fabricated by water-bath pulling with different concentration of AgNWs/ethanol solution. With the increase of solution concentration, more AgNWs were available to float on the water-air surface, so the arrangement of AgNWs on the substrate would be tighter and tighter, as shown in **Figure S3k**. In this experiment, the volume of solution was controlled at 0.3 ml and the water temperature was 40 °C.

Volume of AgNWs Solution

The volume of AgNWs/ethanol solution has the same effect on the interval between the adjacent AgNWs as the concentration does, so the increase of solution volume contributes much to the tight arrangement of AgNWs on the substrate, as shown in **Figure S3g** to **S3i**. However, when too much AgNWs solution was dropped, the water-air surface was saturated with AgNWs, resulting in that when they were transferred to PDMS substrate, AgNWs turned out to be touched with each other and the interval remained unchanged if further increasing the solution volume. Here, we conducted the experiment with 0.4 wt.% AgNWs solution under 60 °C.

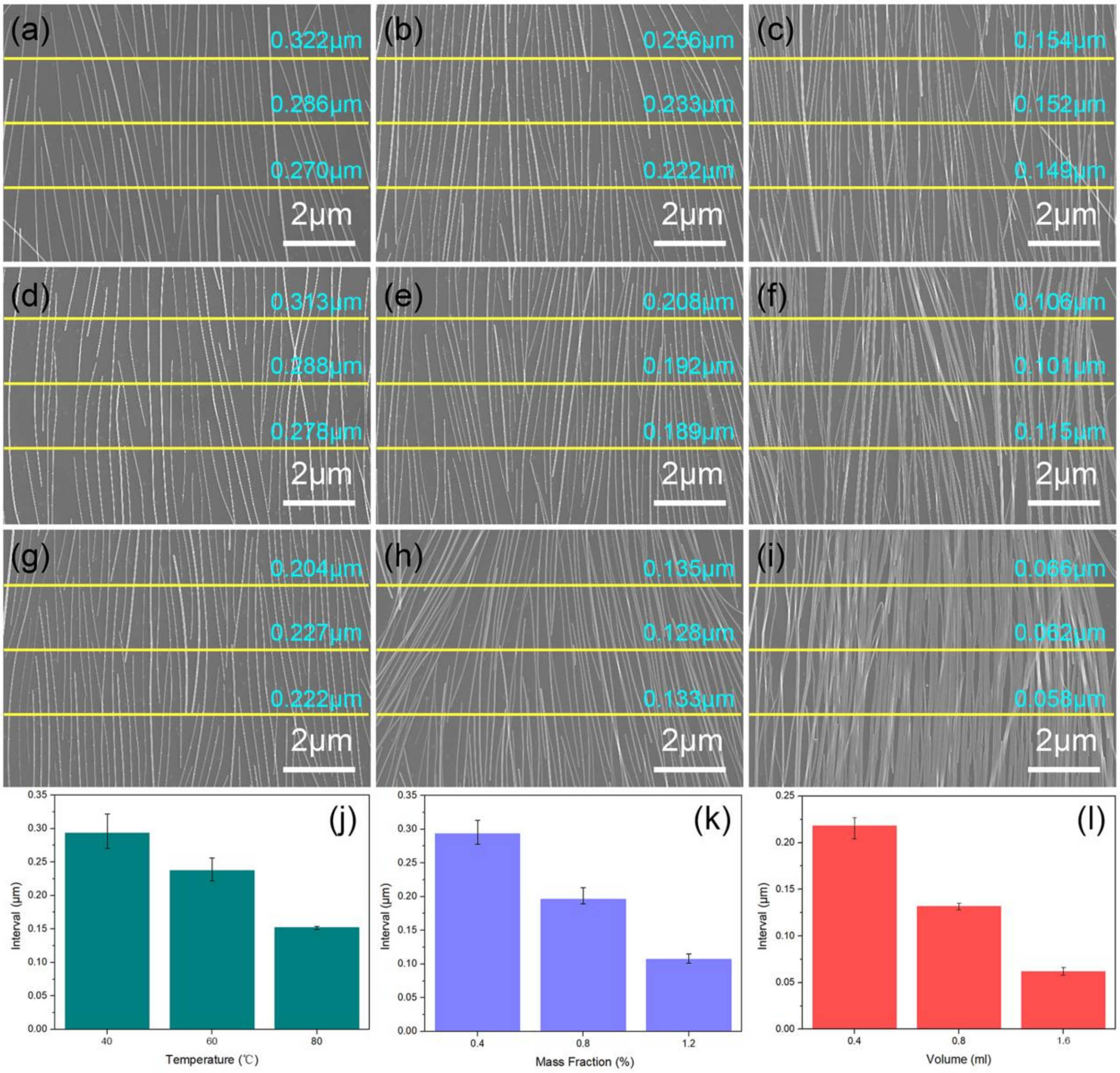

Figure S3 SEM images of aligned AgNWs on the substrate under different water-bath pulling conditions: Water temperature: (a) 40 °C, (b) 60 °C, (c) 80 °C. AgNWs concentration: (d) 0.4 wt.%, (e) 0.8 wt.%, (f) 1.2 wt.%. Volume of solution: (g) 0.4 ml, (h) 0.8 ml, (i) 1.6 ml. Interval cartogram of AgNWs under different water-bath pulling conditions: (j) Water temperature, (k) AgNWs concentration, (l) Volume of solution.
